# Ultrasound elastography score and strain index in different parathyroid lesions

**DOI:** 10.1530/EC-19-0443

**Published:** 2019-11-19

**Authors:** Bekir Cakir, F Neslihan Cuhaci Seyrek, Oya Topaloglu, Didem Ozdemir, Ahmet Dirikoc, Cevdet Aydin, Sefika Burcak Polat, Berna Evranos Ogmen, Ali Abbas Tam, Husniye Baser, Aylin Kilic Yazgan, Mehmet Kilic, Afra Alkan, Reyhan Ersoy

**Affiliations:** 1 Department of Endocrinology and Metabolism, Faculty of Medicine, Ankara Yildirim Beyazit University, Ankara, Turkey; 2 Department of Pathology, Ankara Ataturk Education and Research Hospital, Ankara, Turkey; 3 Department of General Surgery, Faculty of Medicine, Ankara Yildirim Beyazit University, Ankara, Turkey; 4 Department of Biostatistics, Faculty of Medicine, Ankara Yildirim Beyazit University, Ankara, Turkey

**Keywords:** atypical parathyroid adenoma, ultrasound elastography, elastography score, parathyroid adenoma, strain index

## Abstract

**Background:**

Despite significant improvement in imaging quality and advanced scientific knowledge, it may still sometimes be difficult to distinguish different parathyroid lesions. The aims of this prospective study were to evaluate parathyroid lesions with ultrasound elastography and to determine whether strain index can help to differentiate parathyroid lesions.

**Methods:**

Patients with biochemically confirmed hyperparathyroidism and localised parathyroid lesions in ultrasonography were included. All patients underwent B-mode US and USE examination. Ultrasound elastography scores and strain index of lesions were determined. Strain index was defined as the ratio of strain of the thyroid parenchyma to the strain of the parathyroid lesion.

**Results:**

Data of 245 lesions of 230 patients were analysed. Histopathologically, there were 202 (82.45%) parathyroid adenomas, 26 (10.61%) atypical parathyroid adenomas, and 17 (6.94%) cases of parathyroid hyperplasia. Median serum Ca was significantly higher in atypical parathyroid adenoma patients than parathyroid hyperplasia patients (*P* = 0.019) and median PTH was significantly higher in APA compared to PA patients (*P* < 0.001). In 221 (90.2%) of the parathyroid lesions, USE score was 1 or 2. The median SI of atypical parathyroid adenomas was significantly higher than parathyroid adenomas and hyperplasia lesions (1.5 (0.56–4.86), 1.01 (0.21–8.43) and 0.91 (0.26–2.02), respectively, *P* = 0.003).

**Conclusion:**

Our study revealed that SI of parathyroid lesions as well as serum calcium, parathyroid hormone levels, and B-mode US features may help to predict the atypical parathyroid adenoma. Ultrasound elastography can be used to differentiate among parathyroid lesions and guide a surgical approach.

## Introduction

Primary hyperparathyroidism (PHPT), one of the most common endocrine disorders and the most common cause of hypercalcaemia (1, 2), is characterised by excessive parathyroid hormone (PTH) secretion, which leads to increased serum calcium (Ca) levels (3). Accordingly, 85–90% of PHPT cases are caused by parathyroid adenomas (PAs), a type of benign tumour (4), with other causes including double adenoma (4%), multiple gland hyperplasia (6%) and parathyroid carcinoma (<1%) (5).

Regardless of aetiology, surgery has been the definitive and curative treatment for PHPT (6). With the recent increase in the use of minimally invasive parathyroidectomy (MIP), many patients with PHPT who have a solitary adenoma undergo unilateral MIP (2). Given that MIP has become the preferred approach in many centres, preoperative localisation of PAs has also become essential (7).

Preoperative localisation of PAs has been primarily achieved through ultrasonography (US) and technetium-99m-sestamibi scintigraphy (MIBI) with or without single photon emission CT (2, 8, 9, 10). Although normal parathyroid glands are not visible through US (11), pathological parathyroid tissues become visible due to the enlargement or altered gland echogenicity (6). Moreover, vascular flow imaging has been used to increase the sensitivity and specificity of US (6). A recent study on 4D-CT, another new modality (2), reported a distinct vascularity pattern, that is the polar vessel sign, in nearly two-thirds of surgically confirmed PAs (12). Despite the significantly improved imaging quality and advanced scientific knowledge, distinguishing between different parathyroid lesions or between parathyroid lesions and other neck lesions, such as cervical lymph nodes and thyroid nodules, may occasionally remain difficult (7, 13).

Ultrasound elastography (USE) is a non-invasive and dynamic technique that objectively evaluates tissue hardness by measuring tissue elasticity (14, 15, 16). The principle for USE is based on the higher likelihood of softer tissues to deform under compression by an external force compared with harder tissues (17). Parathyroid adenomas are expected to be firm due to decreased fat tissues in PAs and thickened capsules (7). Indeed, studies have shown that parathyroid USE can be helpful for preoperative localisation of PAs among patients with PHPT (2, 7, 18, 19). The first study evaluating focal parathyroid gland lesions using real-time USE by Unluturk *et al*. published in 2012 (7) showed that PAs were elastographically firm lesions and that almost half of the parathyroid hyperplasia lesions were, by contrast, soft during elastographic evaluation (7).

Previous studies have compared USE findings for PAs with those for parathyroid hyperplasia, thyroid nodules or lymph nodes among patients with hyperparathyroidism. The present prospective study aimed to evaluate the use of USE in different parathyroid pathologies (PA, atypical PAs (APAs) and hyperplasia) and determine whether the strain index (SI) can be used to differentiate between such lesions. To the best of our knowledge, this has been the largest study to utilise USE to evaluate parathyroid lesions and the first to utilise USE for APAs.

## Materials and methods

This prospective, single-centre study was approved by the Ethics Committee of Yildirim Beyazit University, Faculty of Medicine. The study protocol followed the tenets of the 1964 Declaration of Helsinki. Consent had been obtained from each patient after full explanation of the purpose and nature of all procedures used.

### Patients

This study recruited patients diagnosed with hyperparathyroidism between January 2016 and January 2019.

The inclusion criteria were as follows: patients older than 16 years, biochemically confirmed hyperparathyroidism and parathyroid lesion localisation in B-mode US. The exclusion criteria were as follows: history of thyroid or parathyroid surgery, percutaneous interventions or radiotherapy within the head and neck region, presence of comorbid diseases (e.g. cardiovascular or respiratory system diseases) contraindicating surgery, patient refusal of surgery and unfavourable pathological results (histopathological diagnosis suggested a lymph node in one patient). Patients selected for follow-up by our multidisciplinary council consisting of surgeons, endocrinologists and nuclear medicine specialists were also excluded.

### Laboratory examinations

Biochemical data immediately prior to surgery, including serum Ca (mg/dL), albumin (g/dL), phosphorus (P) (mg/dL), PTH (pg/mL), alkaline phosphatase (ALP) (IU/L), 25-hydroxyvitamin D (µg/L), creatinine (Cr) (mg/dL) and 24-h urinary Ca (uCa) (mg/day) and P (uP) (g/day) excretion, were obtained from the patients’ records. Total calcium was determined using a reference clinical chemistry laboratory (8.5–10.5 mg/dL) (Roche Diagnostics). Plasma intact PTH was measured using the Allegro IRMA (Roche Diagnostics) with a detection limit of 1 pg/mL (normal range, 15–60 pg/mL) and a 2 and 10% intra- and inter-assay coefficient of variation, respectively. Vitamin D was measured using liquid chromatography coupled with tandem mass spectrometry (Schimadzu-API LC-MS-MS API 3200, Canada) with lower and upper detection limits of 4 and 150 μg/L (normal range, 20–80 μg/L), respectively. Reference ranges for albumin, P, ALP, Cr, 24-h uCa and 24-h uP excretion were 3.5–5.2 g/dL, 2.5–4.5 mg/dL, 36–113 IU/L, 0.5–1.1 mg/dL, 25–300 mg/day and 0.4–1.3 g/day, respectively. In addition, dual-energy X-ray absorptiometry and renal US were performed to evaluate bone mineral density (BMD) and nephrolithiasis, respectively.

### Conventional ultrasonography

An Esaote Colour Doppler system (Model 796FDII; MAG Technology Co. Ltd., Yung-Ho City, Taipei, Taiwan) with a superficial probe (Model LA523 13e4, 5.5e12.5 MHz) was used for US. The procedure was applied to patients in the supine position with their necks hyperextended and skin coated with acoustic material. During B-mode US, parathyroid lesion features, such as size, volume, neck location, echogenicity (hypoechogenic, hyperechogenic and isoechogenic), texture (solid and partial cystic) and vascularisation pattern (type 0, absent; type 1, peripheral; type 2, branching into the adenoma; and type 3, intraparathyroidal) were evaluated.

### Ultrasound elastography

A Hitachi EUB 7500 model elastography (Hitachi Medical Corporation 4-14-1) and a superficial probe (13–5: EUP-L54MA 5–13 MHz, Hitachi Medical Corporation 4-14-1) compatible therewith were used for USE. Given that real-time tissue USE and two properties (USE score and SI) had already been used in our previous studies (15, 17, 20), we opted to utilised such methods in the present study. None of the researchers involved in this study were members of or consultants for Hitachi Medical, General Electric, MAG Technology, or any other company that manufactures a US machine featuring USE.

Free-hand compression applied to the neck was standardised according to real-time USE measurements and presented as a numerical grading scale ranging from 1 to 5. Accordingly, the optimal compression level during USE evaluation was grade 3–4. During USE, the examiner applied recurrent compression on a selected area using the US probe. All images were stored and reviewed subsequently.

**Figure 1:**
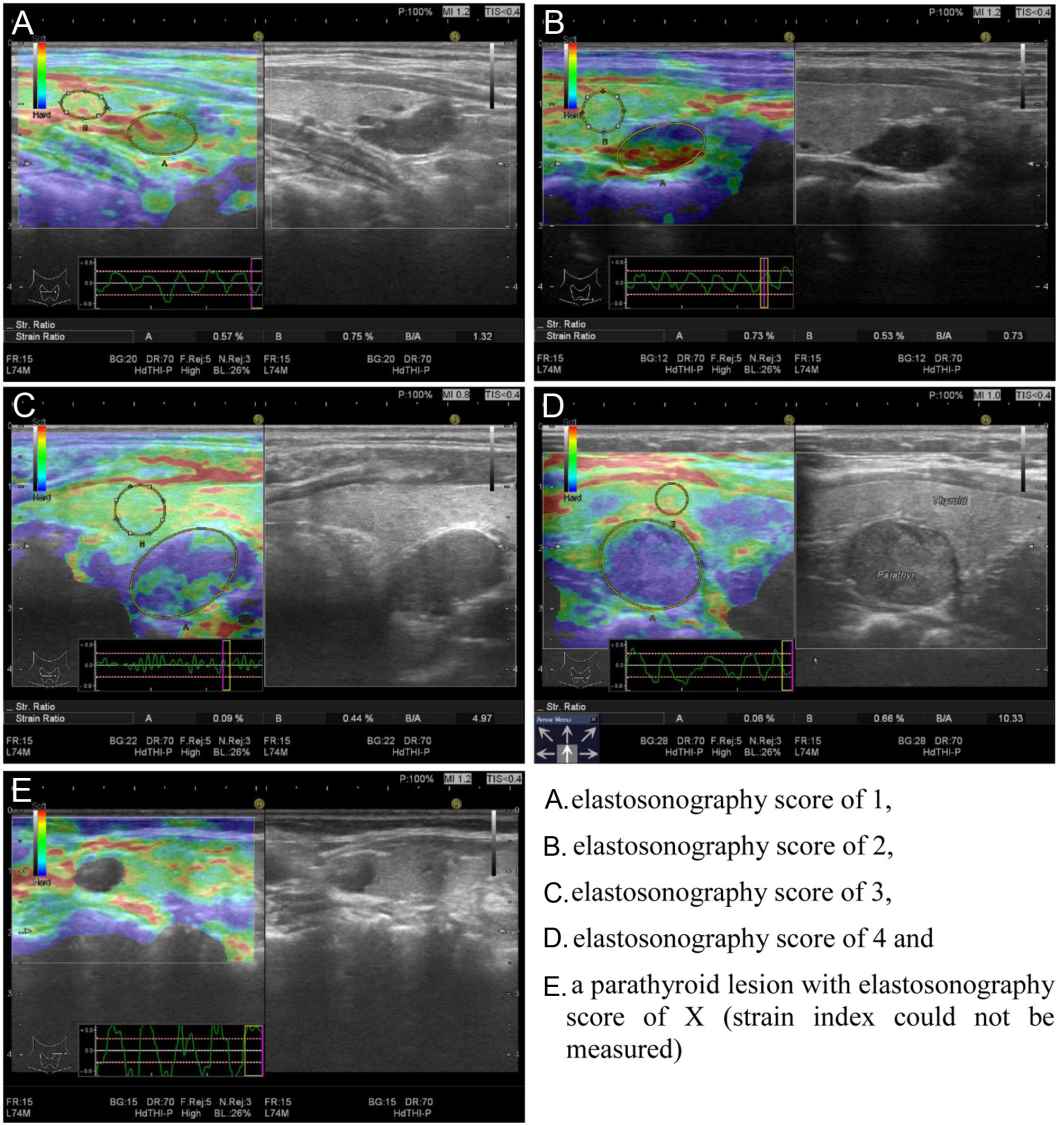
Ultrasound elastography score and strain index measurements in parathyroid lesions.

The obtained USE images were matched using a colour scale and classified using the elasticity score developed by Itoh *et al*. and modified by Asteria *et al*. (21, 22), which assigns lesions a score between 1 and 4. Accordingly, the elastography superimposes information on B-mode images in a colour scale depending on the magnitude of the strain: red (soft tissue), green (intermediately firm tissue) and blue (anelastic tissue) (22). Furthermore, an elastography score of 1 indicates that elasticity extends over the whole examined area with a homogeneously green tumour; a score of 2 indicates that elasticity extends over a large portion of the examined area with almost the entire tumour being light green with some peripheral and/or central blue areas; a score of 3 indicates no elasticity over a large portion of the examined area with almost the entire tumour being hard blue with some light green and red areas; and a score of 4 indicates no elasticity over the entire examined area and with the tumour being homogenously hard blue (22). Lesions that had no colour or were incompressible were indicated with ‘score X’ (Fig. 1).

After scoring, parathyroid lesions were marked, and the lesion strain (A) was determined. Subsequently, a similar sized area of adjacent thyroid tissue was selected, and the strain of this parenchyma (B) was determined. The SI was defined as the ratio between thyroid parenchyma and parathyroid lesion (B/A) strains and calculated automatically using software. For each lesion, strain measurements and SI calculations were performed four times using four consecutive images, the average value of which was recorded as the final SI. All measurements were performed by an experienced endocrinologist (B.Ç).

Given that atrophic thyroiditis can affect the strain, patients who had such a disease upon US were excluded from strain analysis. Moreover, patients with thyroid nodules were measured using the extra-nodular parenchyma.

### Technetium-99m-sestamibi scintigraphy

Parathyroid scanning was performed using intravenously injected 15 mCi Technetium-99m-methoxy-isobutyl-isonitrile (sestamibi). Anterior static images of the neck and mediastinum were then obtained 10 min and 3 h after the injection. At the 3-h time point, CT and/or single photon emission CT images were obtained to confirm the anatomic correlation and attenuation within the neck region. A distinct focus of increased or separate sestamibi uptake relative to the thyroid gland on either early or late images (or both) indicated a positive result.

### Fine-needle aspiration biopsy with parathyroid hormone washout

Patients with suspected parathyroid lesions on US but negative or inconclusive MIBI findings were evaluated using fine-needle aspiration with PTH washout (FNA-PTH). The procedure was performed under US guidance with a 25-gauge needle. After smearing the aspirated material on the slides for cytological examination, the needle was washed out with 500 μL of 0.9% normal saline. A positive FNA‐PTH result was defined as a higher serum PTH level than that upon sampling.

### Statistical analysis

The distribution of continuous variables was determined using Shapiro–Wilk’s test and normality graphs. Continuous and categorical variables were presented as median (min–max) and number (%), respectively.

Kruskal–Wallis and chi-square tests were used to compare continuous and categorical variables between groups, respectively. Dunn–Bonferroni correction was applied in* post hoc* tests. The discriminative ability of PTH, Ca and SI was determined using receiver operating characteristic (ROC) curve analysis, whereas that for MIBI and texture was determined using McNemar’s test. The area under curve (AUC), cut-off point, sensitivity, specificity and their 95% CIs were reported. Wilson’s score method was used to calculate the CIs for sensitivity and specificity. A *P* value less than 0.05 was considered statistically significant.

Wilson’s score CIs were obtained using the ‘scoreci’ function of the ‘PropCI’ library in R ver. 3.5.1 and RStudio ver. 1.2.1335. All other statistical analyses were performed using IBM SPSS Statistics 22.0 (IBM Corp. Released 2012. IBM SPSS Statistics for Windows, version 22.0.: IBM Corp.).

## Results

### Clinical features and preoperative biochemical evaluation

**Figure 2:**
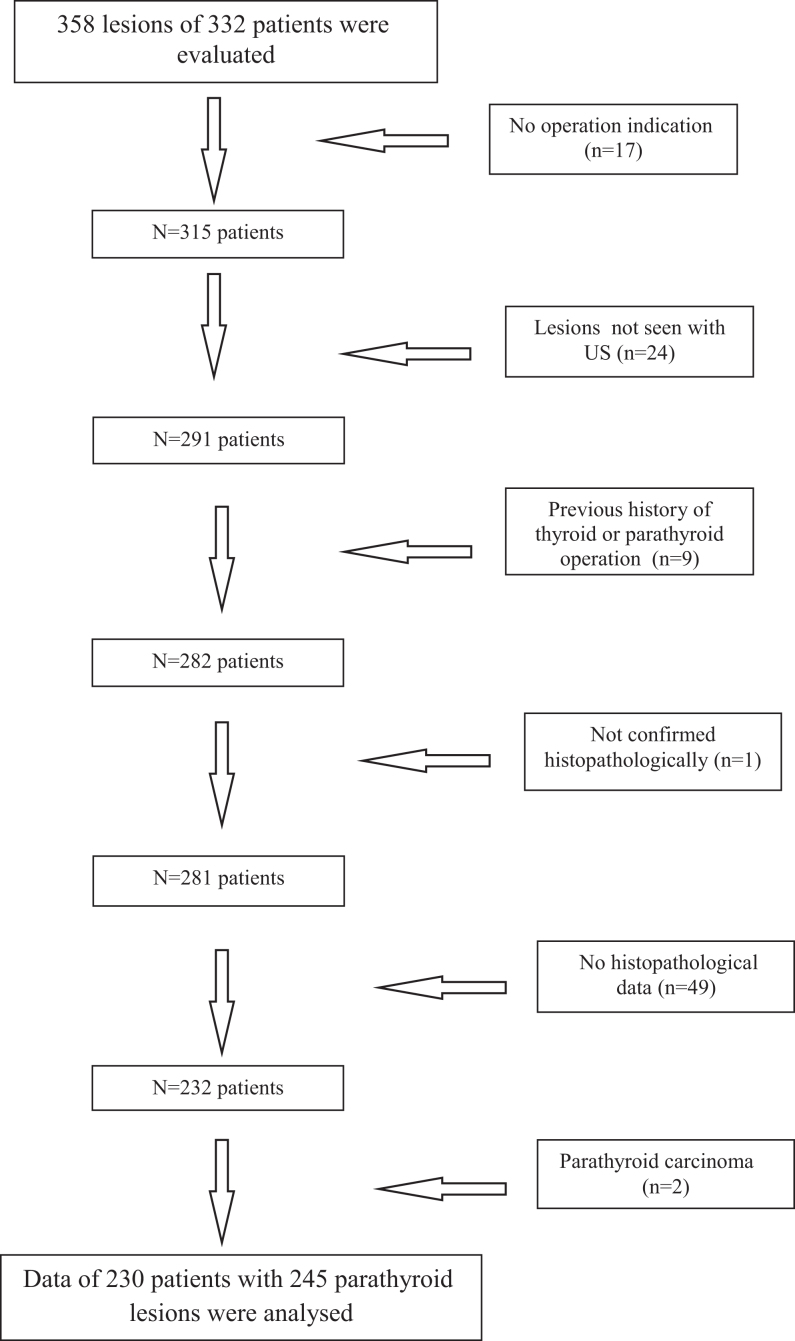
Flowchart of the patients in the study.

A total of 358 parathyroid lesions from 332 patients were evaluated. Our multidisciplinary council determined that 17 patients had no indication for surgery and recommended follow-up. Moreover, US could not determine lesion localisation in 24 patients. Nine patients had a history of thyroid or parathyroid surgery, whereas 49 patients did not undergo surgery at our centre. One patient did not undergo histopathological confirmation of the lesion, whereas two patients had a histopathological diagnosis of parathyroid carcinoma (Fig. 2). After excluding the aforementioned patients, 245 lesions from 230 patients were ultimately analysed.

**Figure 3:**
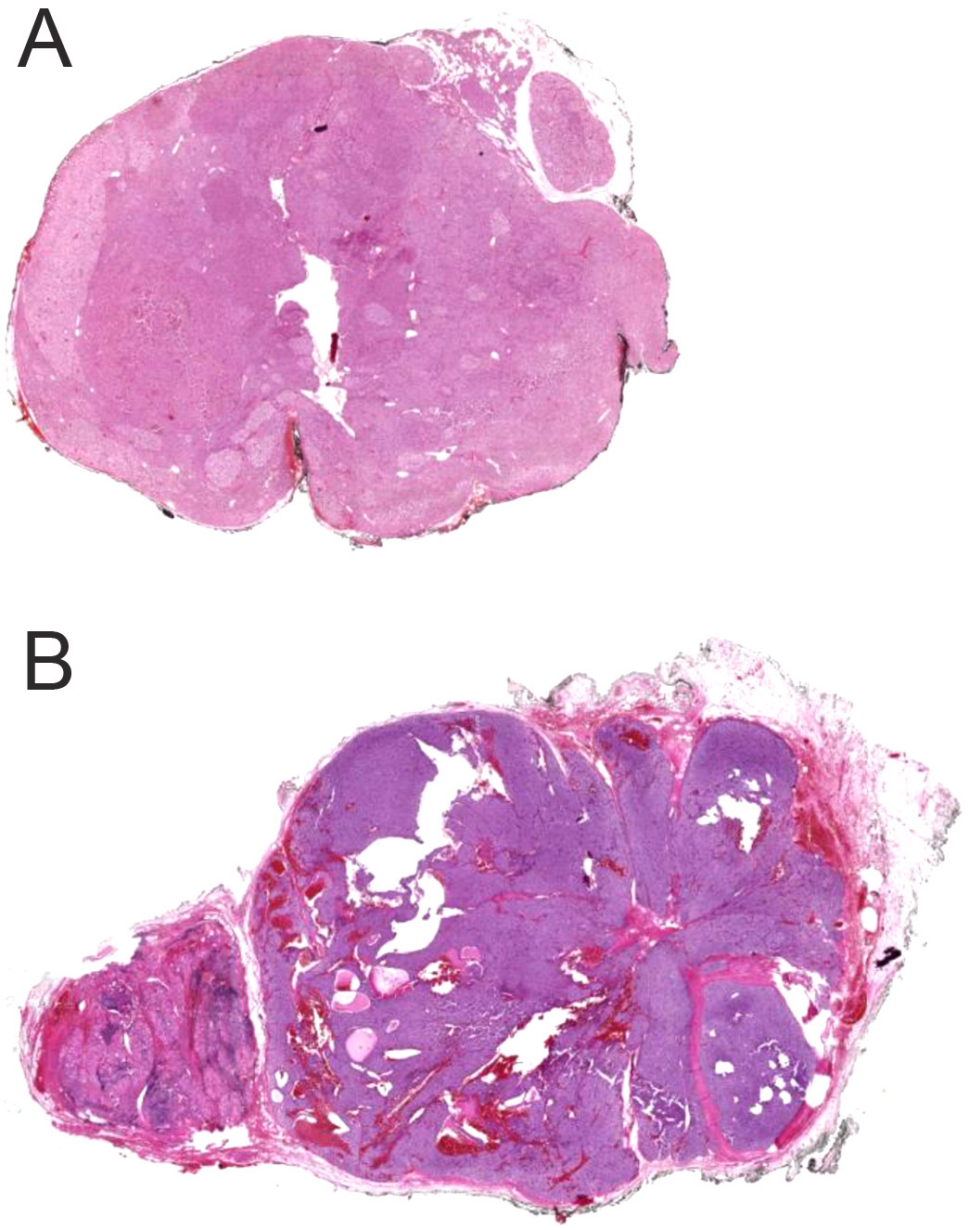
Whole slide imaging (WSI) of the (A) parathyroid adenoma; diffuse pattern of parathyroid adenoma consisting of chief and clear cells with normal parathyroid tissue at one side, (B) atypical parathyroid adenoma; thick fibrous bands between the neoplastic cells, cystic spaces and the presence of neoplastic cells in the capsule.

Among the included patients, 202 (87.8%) and 28 (12.2%) were female and male, respectively, with a median age of 54 (20–82) years. Demographical features and preoperative biochemistry, BMD and urinary US results are presented in Table 1. Histopathologically, 193 patients had PA, 25 had APA (Fig. 3) and 12 had parathyroid hyperplasia. Clinical diagnosis and histopathological findings are presented in Table 2.

**Table 1:** Demographical, clinical and biochemical findings of patients with different parathyroid lesions.

Variables	All patients (*n* = 230) (%)	Hyperplasia (*n* = 12) (%)	Adenoma (*n* = 193) (%)	Atypical adenoma (*n* = 25) (%)	*P*
Age (years)	54 (20–82)	49.5 (26–71)	54 (20–78)	52 (34–82)	0.636
Sex					0.729
Female	202 (87.8)	10 (83.3)	171 (88.6)	21 (84)	
Male	28 (12.2)	2 (16.7)	22 (11.4)	4 (16)	
Calcium (mg/dL)	11.3 (9.2–18.5)	10.8 (10.1–12.1)	11.3 (9.2–18.5)	11.4 (10.2–15.3)	**0.024a**
Albumin (g/dL)	4.6 (3.6–5.8)	4.4 (4.2–4.9)	4.6 (3.6–5.8)	4.6 (3.8–4.9)	0.616
Phosphorus (mg/dL)	2.6 (1.1–7.0)	2.5 (1.8–6.1)	2.6 (1.1–7.0)	2.3 (1.4–3.9)	0.171
Creatinine (mg/dL)	0.7 (0.3–9.5)	0.7 (0.5–9.5)	0.7 (0.3–8.4)	0.7 (0.4–4.2)	0.878
ALP (IU/L)	96 (27–1163)	104 (61–776)	93.5 (32–1163)	117 (27–427)	0.148
PTH (pg/mL)	139 (44–2800)	186 (71.9–2235)	133.8 (44–2800)	209 (93–1703)	**<0.001b**
25-Hydroxy vitamin D (µg/L)	15.8 (2.2–101)	14.4 (7.6–55)	16.0 (2.2–101)	9.4 (3.2–51.7)	0.322
24-h urinary calcium (mg/day)	374.5 (55.5–1191)	400 (152–947)	375 (55.5–1153)	358 (113–1191)	0.929
24-h urinary phosphorus (g/day)	735 (0.8–2060)	850 (490–1320)	740 (0.8–2060)	695 (360–1810)	0.423
BMD	*n* = 203	*n* = 12	*n* = 170	*n* = 21	0.215
Normal	41 (20.2)	0 (0)	36 (21.2)	5 (23.8)	
Osteopenia	65 (32)	5 (41.7)	53 (31.2)	7 (33.3)	
Osteoporosis	97(47.8)	7 (58.3)	81 (47.6)	9 (42.9)	
Nephrolitiasis	*n* = 213	*n* = 9	*n* = 181	*n* = 23	0.117
Present	57 (26.8)	5 (55.6)	45 (24.9)	7 (30.4)	
Absent	156 (73.2)	4 (44.4)	136 (75.1)	16 (69.6)	

^a^*P* = 0.094 for hyperplasia vs adenoma, *P* = 0.019 for hyperplasia vs atypical adenoma, *P* = 0.397 for adenoma vs atypical adenoma. ^b^*P* = 0.461 for hyperplasia vs adenoma, *P* = 0.773 for hyperplasia vs atypical adenoma, *P* < 0.001 for adenoma vs atypical adenoma. Bold indicates *P *< 0.05.

ALP, alkaline phosphatase; BMD, bone mineral density; PTH, parathyroid hormone.

**Table 2:** Clinical diagnosis and histopathological findings of the patients.

Clinical hyper-parathyroidism status	Causative clinical diagnosis	Number of patients with their parathyroid lesions
Primary hyper-parathyroidism (*n* = 226)	Only primary hyper-parathyroidism	213 patients with single adenoma 2 patients has 4 hyperplasia lesions 10 patients have double adenomas
	MEN-1	1 patient with 3 hyperplasia lesions
Tertiary hyper-parathyroidism (*n* = 4)	CRD	1 patient with 1 hyperplasia lesion 1 patient with 2 hyperplasia lesions 2 patients with single adenoma

CRD, chronic renal disease; MEN-1, multiple endocrine neoplasia-1.

### Patient-based comparisons of demographical and clinical characteristics

Median age and sex distribution were similar in all three groups (Table 1). Patients with APA had a significantly higher median serum Ca level compared with those with parathyroid hyperplasia (*P* = 0.019) and a significantly higher median PTH level compared with those with PA (*P* < 0.001). PTH was discriminative only between patients with APA and PA, with an AUC of 0.739 (95% CI: 0.640–0.838), cut-off point of ≥168.5 pg/mL, sensitivity of 72% and specificity of 64.6%. No significant difference in serum albumin, P, ALP, 25-hydroxyvitamin D and 24-h uCa and uP levels were observed between the groups. All groups had similar osteopenia/osteoporosis and nephrolithiasis rates.

### Scintigraphy results and lesion-based comparison of conventional and power Doppler ultrasonography

All groups had similar lesion localisation (Table 3). APAs had greater anteroposterior and transverse diameter and volume compared with PAs and parathyroid hyperplasia lesions. APAs had a significantly greater longitudinal diameter than PAs. Almost all APAs (23/26), most PAs (186/202) and all hyperplasia lesions (17/17) were hypoechoic. No significant difference in blood flow pattern were observed between the lesions (*P* = 0.635). A partial cystic component was significantly more frequent among APAs than PAs (*P* = 0.004). Hyperplasia lesions had significantly lower MIBI positivity rates than PAs and APAs (*P* = 0.047 and 0.011, respectively).

**Table 3:** Conventional ultrasonography and ultrasound elastography features, Technetium-99m-sestamibi results and histopathological diameter of different parathyroid lesions.

	Hyperplasia (*n* = 17) (%)	Adenoma (*n* = 202) (%)	Atypical adenoma (*n* = 26) (%)	*P* value
Side				0.518
Left	9 (52.9)	94 (46.5)	15 (57.7)	
Right	8 (47.1)	108(53.5)	11 (42.3)	
Localization				**–**
Superior	2 (11.8)	18 (8.9)	3 (11.5)	
Middle	3 (17.6)	38 (18.8)	4 (15.4)	
Inferior	11 (64.7)	145 (71.8)	19 (73.1)	
Ectopic	1 (5.9)	1 (0.5)	0 (0.0)	
Diameter (mm)				
Anteroposterior	8.6 (4.5–20.3)^a^	8.8 (2.4–26.5)^b^	11.55 (5.2–30)^ab^	**0.007**
Transverse	8 (3.2–17.4)^a^	7.8 (2.4–22.7)^b^	11.4 (3.5–45)^ab^	**<0.001**
Longitudinal	14 (6.5–28)	14.6 (3.4–48.5)^a^	20.35 (6.9–49.7)^a^	**0.029**
Volume (mL)	0.49 (0.06–3.31)^a^	0.54 (0.04–14.99)^b^	1.51 (0.07–31.6)^ab^	**0.002**
Echogenicity				–
Hypoechoic	17 (100.0)	186 (92.1)	23 (88.5)	
Isoechoic	0 (0.0)	14 (6.9)	3 (11.5)	
Hyperechoic	0 (0.0)	2 (1.0)	0 (0.0)	
Texture				**0.010c**
Solid	14 (82.4)	174 (86.1)	16 (61.5)	
Partial cystic	3 (17.6)	28 (13.9)	10 (38.5)	
Blood flow pattern				0.635
Type 0	0 (0)	24 (11.9)	3 (11.5)	
Type 1	8 (47.1)	77 (38.1)	13 (50.0)	
Type 2	7 (41.2)	69 (34.2	6 (23.1)	
Type 3	2 (11.7)	32 (15.8)	4 (15.4)	
Elastography score				**–**
Score 1	9 (52.9)	84 (41.5)	7 (26.9)	
Score 2	5 (29.4)	100 (49.5)	16 (61.6)	
Score 3	2 (11.8)	9 (4.5)	2 (7.7)	
Score 4	0 (0.0)	1 (0.5)	0 (0.0)	
Score X	1 (5.9)	8 (4.0)	1 (3.8)	
Median strain index	0.91 (0.26–2.02)^a^	1.01 (0.21–8.43)^b^	1.5 (0.56–4.86)^ab^	**0.003**
MIBI	*n* = 17	*n* = 183	*n* = 24	**0.038**^d^
Positive	6 (35.3)	110 (60.1)	18 (75)	
Negative	11 (64.7)	73 (39.9)	6 (25)	
Histopathological diameter (mm)	1.5 (0.4–3.0)^a^	1.5 (0.5–5.5)^b^	2 (1.0–5.0)^ab^	**0.008**

^ab^Groups are significantly different for corresponding measurement (*P* < 0.05). ^c^*P* = 0.710 for hyperplasia vs adenoma, *P* = 0.140 for hyperplasia vs atypical adenoma, *P* = 0.004 for adenoma vs atypical adenoma. ^d^*P* = 0.047 for hyperplasia vs adenoma, *P* = 0.011 for hyperplasia vs atypical adenoma, *P* = 0.160 for adenoma vs atypical adenoma. Bold indicates *P *< 0.05.

Histopathologically, APAs had a significantly higher median diameter than both PAs and parathyroid hyperplasia lesions (*P* = 0.008).

### Lesion-based comparison of elastography results

Among the hyperplasia lesions, PAs and APAs, 9 (52.9%), 84 (41.5%) and 7 (26.9%) had a USE score of 1 (Table 3), whereas 5 (29.4%), 100 (49.5%) and 16 (61.6%) had a USE score of 2, respectively. Among ten lesions with score X, 8 (80.0%) were PAs.

**Figure 4:**
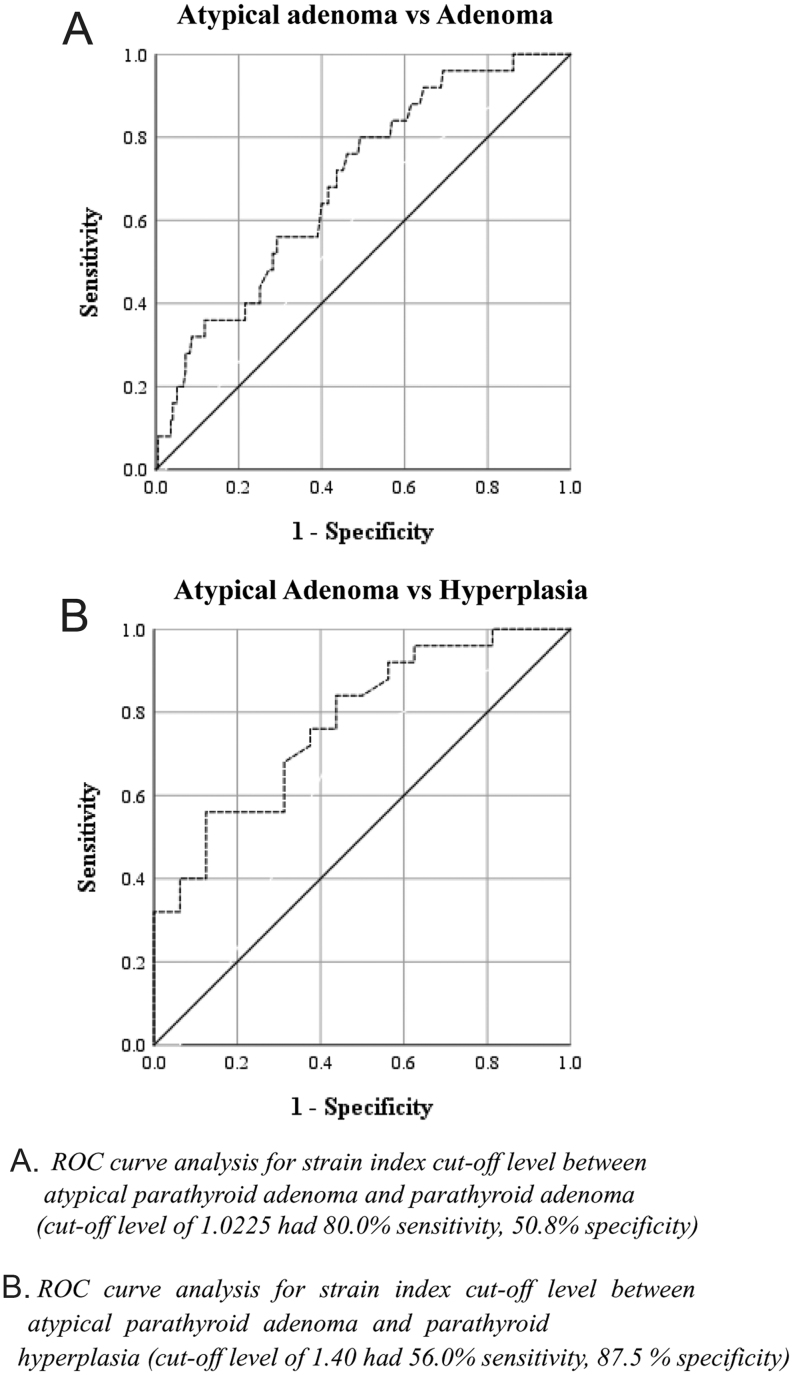
ROC curves for strain index.

Significant differences in the median SI was observed between the groups (*P* = 0.003). Accordingly, APAs had the highest and parathyroid hyperplasia lesions had the lowest SIs (Table 3). ROC curve analysis determined that an SI cut-off level of 1.0225 could discriminate between APAs and PAs with an AUC of 0.69, sensitivity of 80.0% and specificity of 50.8% (Fig. 4 and Table 4). Moreover, the same analysis found that an SI cut-off level of 1.40 was able to discriminate between APAs and parathyroid hyperplasia lesions, with an AUC of 0.77, sensitivity of 56% and specificity of 87.5%.

**Table 4:** Discrimination performance of the strain index in different parathyroid lesions.

	Adenoma vs hyperplasia	Atypical adenoma vs adenoma	Atypical adenoma vs hyperplasia
	(*n* = 194 vs *n* = 16)	(*n* = 25 vs *n* = 194)	(*n* = 25 vs *n* = 16)
Mean SI			
AUC (95% CI)	0.597 (0.458–0.736)	0.690 (0.588–0.792)	0.773 (0.628–0.917)
*P* value	0.198	**0.002**	**0.004**
Cut-off point	–	≥1.0225	≥1.40
Sensitivity (95% CI)	–	0.800 (0.609–0.911)	0.560 (0.371–0.733)
Specificity (95% CI)	–	0.508 (0.438–0.577)	0.875 (0.640–0.965)

AUC, area under curve; SI, strain index. Bold indicates *P *< 0.05.

## Discussion

This prospective study evaluated the diagnostic accuracy of USE scoring and SI in differentiating between parathyroid lesions in addition to B-mode US features and vascularity pattern among patients with hyperparathyroidism. Although patients with PA, APA and parathyroid hyperplasia had similar USE scores, APAs had a significantly higher SI than PAs and parathyroid hyperplasia lesions.

Accurate preoperative localisation of parathyroid lesions has been considered crucial for both safety and efficacy of the surgical approach, particularly for MIP. Accordingly, US has been the most widely used method for parathyroid lesion localisation. Conventional US cannot detect a normal parathyroid gland given its small size, deep positioning, similarity in structural pattern to normal thyroid parenchyma and fat tissue component making detection more difficult (7). However, parathyroid gland enlargement allows visualisation through US. Indeed, studies have reported that this method had a sensitivity and specificity of 69–90% and 90–98% for enlarged parathyroid gland localisation, respectively (23). High-resolution grey scale images, power Doppler US showing vascular flow imaging and examiner experience may increase the sensitivity of US (3). Moreover, the combined use of US and MIBI had been found to increase the sensitivity of these methods to 95% in enlarged parathyroid lesions (24).

Despite considerable advancements in imaging techniques, distinguishing between different parathyroid lesions or between parathyroid lesions and other neck lesions may occasionally remain difficult (7, 13), with false-positive results usually being derived from thyroid nodules (6–15%) (19) or enlarged lymph nodes (6). Moreover, US has certain limitations, such as its low sensitivity in detecting focal ectopic or atypically located parathyroid tissues and smaller lesions (6, 19). Additionally, examiner knowledge and experience can be considered important contributors to the diagnostic utility of this method (6).

As mentioned previously, USE, which evaluates tissue hardness, has been found to be useful in the preoperative localisation of PAs. A normal parathyroid gland is composed of chief cells, fibrovascular stroma and 70% fat tissue (3, 18). However, the ratio of fat tissue variably decreases in PAs. One study showed that the characteristic hypoechoic appearance of PAs on US is due to its hypercellular internal structure with a low fat component (3). As expected, the present study found that almost all APAs, most PAs and all parathyroid hyperplasia lesions were hypoechoic. Studies have also shown that PAs have hard and fibrous capsules (3, 18). The main principle for USE is based on the concept that applying external compression to the tissue causes lesser strain (longitudinal tissue replacement) in harder tissues than in softer ones (25, 26, 27, 28, 29, 30, 31, 32). Only a few studies have used USE among patients with parathyroid lesions. Accordingly, a literature search identified only ten studies evaluating the elasticity of parathyroid lesions with different USE techniques (Table 5). The first USE study by Unluturk *et al*. showed that PAs were significantly stiffer (determined using SI) than parathyroid hyperplasia lesions (3.56 vs 1.49, respectively) (7). The present study found that APAs had a significantly higher SI than both PAs and parathyroid hyperplasia lesions (1.5, 1.01 and 0.91, respectively). Consistent with the results presented herein, Hattapoglu *et al*. (18) revealed that PAs had a significantly higher mean shear wave velocity than parathyroid hyperplasia lesions (2.28 ± 0.5 and 1.46 ± 0.23 m/sn, respectively). However, the major limitations of their study included the presence of few parathyroid hyperplasia cases and the use of the Virtual Touch Tissue Quantification technique, which is difficult to utilise in small lesions.

**Table 5:** Published studies evaluating parathyroid lesions with ultrasound elastography.

Study	PA (*n*)	PH (*n*)	PC (*n*)	Thyroid nodule/tissue (*n*)	Lymph node (*n*)	ESG technique and medical company	Main finding
Azizi * et al.* (2)	57	–		54	–	SWE using the virtual touch imaging quantification Siemens	Median shear wave velocity was 2.02 m/sn in PAs, whereas 2.77 m/sn in thyroid tissue
Polat * et al.* (3)	54	33			31	SWE using the virtual touch tissue imaging quantification Siemens	Mean shear wave velocity of PAs were higher than PHs or lymph nodes (2.16 ± 0.33 m/sn, 1.75 ± 0.28 m/sn and 1.86 ± 0.37 m/sn, respectively)
Altinbas and Yagci (4)	46	4			52 lymph nodes in HT patients and 51 juguler reactive lymph nodes	Elasticity score measured by real time strain sonoelastography GE Healthcare	Elasticity score of parathyroid lesions, lymph nodes of HT patients and reactive jugular lymph nodes were; 2.30 ± 0.91, 2.70 ± 0.93 and 1.88 ± 0.59, respectively
Unluturk * et al.* (7)	59	32		–	–	Strain ratio Hitachi	Median strain ratio of PAs were higher than PHs (3.56 vs 1.49)
Golu * et al.* (14)	21	1		43	–	Elasticity index measured by 2D-SWE Supersonic Imagine	Mean elasticity index was lower in parathyroid lesions than thyroid parenchyma (10.2 ± 4.9 vs 19.5 ± 7.6 kPa)
Stangierski * et al.* (16)	65			51		Elasticity measured by SWE Supersonic Imagine	PAs were more elastic than benign thyroid nodules (mean elasticity were 5.2 ± 7.2 vs 24.3 ± 33.8 kPa)
Hattapoğlu * et al.* (18)	32	4		21/36		SWE using virtual touch tissue quantification method Siemens	The mean shear wave velocity of PAs, PHs, benign thyroid nodules and normal thyroid parenchyma were 2.28 ± 0.5 m/sn, 1.46 ± 0.23 m/sn, 2.25 ± 0.51 m/sn and 1.62 ± 0.20, respectively
Batur * et al.* (19)	21			71		2D-SWE using acoustic radiation force impulse imaging Siemens	PAs had significantly higher stiffness than benign thyroid nodules and lower stiffness than malignant thyroid nodules (mean shear wave velocity was 3.09 ± 0.75, 2.20 ± 0.39 and 3.59 ± 0.43 m/sn, respectively)
Isidori * et al.* (24)	15	29	3	18	14	Elastoscan core index with ultrasound elastography Samsung	Mean elastoscan core index of PAs were higher than PHs and reactive lymph nodes (1.77 ± 0.54, 1.35 ± 0.61, 1.12 ± 0.58, respectively) and similar to benign thyroid nodules (1.72 ± 0.66) and lower than PCs (3.47 ± 0.77)
Chandramohan * et al.* (31)	43	4		93		SWE using acoustic radiation force impulse technology. Shear wave velocity measured with virtual touch quantification Siemens	The mean shear wave velocity of the parathyroid lesion (1.6 ± 0.78 m/s) was significantly lower than benign (2.11 ± 0.8 m/s) and malignant (4.3 ± 2.71 m/s) thyroid nodules

HT, hashimoto thyroiditis; PA, parathyroid adenoma; PC, parathyroid carcinoma; PH, parathyroid hyperplasia; SWE, shear wave elastography.

The present study revealed that 41.5, 26.9 and 52.9% of PA, APA and hyperplasia lesions, respectively, had a USE score of 1. Moreover, slightly more PAs and APAs than parathyroid hyperplasia lesions had a USE score of 2. Accordingly, histopathological differences may partly explain the higher degree of stiffness in PAs compared with hyperplasia lesions on USE. Indeed, studies have shown that although a significant decrease in fat tissue ratio had been observed for PAs, only a slight decrease thereof had been observed for parathyroid hyperplasia (3, 7, 18). In addition, capsule thickening has been more frequently observed in PAs than in parathyroid hyperplasia lesions (25).

Given the absence of studies evaluating APA using USE, we could not compare our findings on APAs. Moreover, the clinical importance and long-term outcomes of these lesions have not been well defined due to the overall low prevalence and lack of a standard definition (26). Studies have suggested that APAs may precede carcinoma development (26) perhaps because they possess some features inherent to carcinomas, such as fibrous band formation, mitotic activity, trabecular growth, tumour adherence to adjacent soft tissues or thyroid tissues and lesional cell entrapment into the surrounding capsule, but do not exhibit evidence of invasive growth (27, 28). No single biochemical or imaging modality can be used to differentiate APAs and parathyroid carcinomas from classic adenomas (26). The present study found that APAs had higher Ca and PTH levels than parathyroid hyperplasia lesions and PAs, respectively. Studies have shown that patients with APA usually have intermediate Ca levels that fall between adenomas and carcinomas (27, 28). In our study, although not statistically significant, patients with APA had lower vitamin D levels than those with PA and parathyroid hyperplasia, which may have contributed to the higher PTH levels found among patients with APA. Additionally, we found that APAs had higher anteroposterior and transverse diameters than PAs and parathyroid hyperplasia lesions, as well as a higher longitudinal diameter than PAs, a result consistent with those presented in our previous study (26). We also found that more APAs than PAs had a cystic component, which is concordant with results presented in the literature (26).

Parathyroid lesions also need to be differentiated from other neck lesions, such as thyroid nodules and lymph nodes (14). After evaluating 2D shear wave USE features of pathologically confirmed PAs, Golu *et al*. (14) revealed that PAs had a lower elasticity index compared with thyroid tissue, which contradicted the findings of Unluturk *et al*. (7). Accordingly, Golu *et al*. (14) attributed this difference to the use of different USE techniques. Chandramohan *et al*. (31) reported that PAs were softer than benign and malignant thyroid nodules, whereas Batur *et al*. (19), who compared 21 PAs with 71 thyroid nodules, found that PAs were stiffer than benign thyroid nodules but softer than malignant ones. Another study involving 57 patients (2) showed that PAs were softer than thyroid tissue. Moreover, Isodiri *et al*. (24) who evaluated 47 parathyroid lesions, 18 ectopic thyroid nodules and 14 reactive lymph nodes using quasi-static USE reported that PAs had a higher mean Elastoscan Core Index value than parathyroid hyperplasia lesions and reactive lymph nodes. After comparing 65 PAs with 51 benign thyroid nodules, Stangierski *et al*. (16) found that the former were more elastic than the latter.

Differentiating PAs from cervical lymph nodes has remained difficult. Accordingly, two studies (2, 32) evaluating different USE techniques revealed that PAs have a lower shear wave velocity than benign lymph nodes. However, studies haves shown that malignant lymph nodes were significantly stiffer than PAs (33, 34, 35). Polat *et al*. (3) found that PAs had a stiffer structure than parathyroid hyperplasia lesions and reactive lymph nodes. Moreover, the study by Altinbas & Yagci (4) showed that parathyroid lesions, lymph nodes of patients with Hashimoto’s disease and jugular lymph nodes of healthy subjects had elasticity scores of 2.3 ± 0.91, 2.7 ± 0.93 and 1.88 ± 0.59, respectively (*P* < 0.05). However, given the small number of parathyroid hyperplasia lesions (*n* = 4, 8%), the aforementioned study could not establish a comparison between PA and parathyroid hyperplasia lesions.

Some limitations of the present study need to be considered. First, several patients had thyroid nodules, which can affect strain measurement. However, to prevent possible confounding effect of nodules, the strain of the thyroid parenchyma was measured from the extra-nodular area. Another limitation of the present study was the use of strain USE. Accordingly, this technique obtains data through transducers during manual tissue compression, which makes it operator dependent. Nevertheless, our operator is highly experienced with the USE technique and has performed valuable work in this field (15, 17, 20). In addition, incompressible or uncoloured lesions could not be evaluated for strain ratio. Lastly, given the single-centre design of the present study, multi-centre studies are required to confirm our findings.

Some strengths of the present study are also worth noting. To the best of our knowledge, no other large study has evaluated different parathyroid lesions (PA, parathyroid hyperplasia and APA) using USE. Moreover, our literature search determined that a total of 523 parathyroid lesions had been evaluated using strain or shear wave USE in 10 studies, whereas 245 parathyroid lesions had been evaluated in the present study alone. Furthermore, all parathyroid lesions analysed herein were surgically excised and histopathologically confirmed.

## Conclusions

The current study revealed that the USE score and SI can help preoperatively identify parathyroid lesions. Considering that APAs have been suggested as a pre-cancerous lesion of the parathyroid gland, differentiating APAs from PAs and parathyroid hyperplasia lesions is imperative. Accordingly, our findings revealed that strain USE measurement of parathyroid lesions, as well as serum Ca and PTH levels, some B-mode US features (diameter, volume, hypoechogenicity and cystic component) and MIBI positivity, may help differentiate between APAs, PAs and parathyroid hyperplasia lesions.

## Declaration of interest

The authors declare that there is no conflict of interest that could be perceived as prejudicing the impartiality of the research reported.

## Funding

This work did not receive any specific grant from any funding agency in the public, commercial, or not-for-profit sector.
